# The Application of MSCs-Derived Extracellular Vesicles in Bone Disorders: Novel Cell-Free Therapeutic Strategy

**DOI:** 10.3389/fcell.2020.00619

**Published:** 2020-07-22

**Authors:** Shuyu Liu, Xia Xu, Shujing Liang, Zhihao Chen, Yan Zhang, Airong Qian, Lifang Hu

**Affiliations:** ^1^Laboratary for Bone Metabolism, Key Lab for Space Biosciences and Biotechnology, School of Life Sciences, Northwestern Polytechnical University, Xi’an, China; ^2^Xi’an Key Laboratory of Special Medicine and Health Engineering, School of Life Sciences, Northwestern Polytechnical University, Xi’an, China; ^3^Research Center for Special Medicine and Health Systems Engineering, School of Life Sciences, Northwestern Polytechnical University, Xi’an, China; ^4^NPU-UAB Joint Laboratory for Bone Metabolism, School of Life Sciences, Northwestern Polytechnical University, Xi’an, China

**Keywords:** mesenchymal stem cells, extracellular vesicles, exosomes, osteoarthritis, rheumatoid arthritis, osteoporosis, bone fracture

## Abstract

Bone is crucial for supporting the body, protecting other organs, providing minerals, and secreting hormone to regulate other organ’s function. Bone disorders result in pain and disability, severely affecting human health, reducing the quality of life and increasing costs to society. With the rapid increase in the aging population worldwide, bone disorders have become one major disease. As a result, efficacious therapies of bone disorders have become the focus of attention worldwide. Mesenchymal stem cells (MSCs) have been widely explored as a new therapeutic method for numerous diseases. Recent evidence suggests that the therapeutic effects of MSCs are mainly mediated by their extracellular vesicles (EV). MSCs-derived extracellular vesicles (MSCs-EV) is indicated as a novel cell-free alternative to cell therapy with MSCs in regenerative medicine. Here, we review the current knowledge of EV and highlight the application studies of MSCs-EV in bone disorders by focusing on osteoarthritis (OA), rheumatoid arthritis (RA), osteoporosis (OP), and bone fracture. Moreover, we discuss the key issues and perspectives of MSCs-EV as a clinical therapeutic strategy for bone diseases.

## Introduction

Bone, one most important supportive structure of human body, plays important roles in supporting body, protecting other organs, providing minerals and secreting hormone to regulate other organs. Bone disorders have a serious impact on human health and have become common diseases among the elderly. Therefore, appropriate and effective treatment of bone diseases is one of the greatest concerns in the world today.

Mesenchymal stem cells (MSCs) are multipotent fibroblast-like cells with the potential for self-renewal and multilineage differentiation. Moreover, MSCs possess the ability to migrate to the damaged sites, where they secret anti-inflammatory factors and growth factors to promote wound healing. Thus, MSCs have become a desirable cell source in regenerative medicine and immune therapy. MSCs transplant treatment (MSCT) is considered to be a promising therapy for a variety of human diseases and has drawn increasing attention. However, the direct use of MSCs for treating diseases faces many challenges, including genetic instability (Yoon et al., 2004; Tolar et al., 2007; Jeong et al., 2011), loss of function (Pak et al., 2003), pathogenicity (Furlani et al., 2009), and limited cell survival (Breitbach et al., 2007).

Recent evidence suggest that MSCs exert their therapeutic capabilities primarily through paracrine secretion of particles, rather than a cellular manner (Wang et al., 2015; Cosenza et al., 2017; Zhou Y. et al., 2019). The secreted particles are collectively referred to as extracellular vesicles (EV), which are usually classified as exosomes, microvesicles (MV), and apoptotic bodies based on different biogenesis way (Yáñez-Mó et al., 2015). Due to the lack of consensus on specific markers for EV subtypes, ISEV recommends the usage of EV, a collective term covering various EV subtypes (Thery et al., 2018). MSCs-derived extracellular vesicles (MSCs-EV) emerge as critical mediators of paracrine effects, with increasing evidence for their function in MSCs-mediated regeneration and immunotherapy (Li et al., 2013; Xin et al., 2013; Tan et al., 2014; Zhao et al., 2018; Biswas et al., 2019; Zhang S. et al., 2019). MSCs-EV regulate the function of recipient cells by transmitting information carried by lipids, nucleic acids and proteins, which are the main components of MSCs-EV (Yáñez-Mó et al., 2015). Compared with MSCT, MSCs-EV treatment show advantages of higher security, convenient storage, transportation and administration (Jing et al., 2018). Therefore, MSCs-EV has received increasing attention for its promising clinical application (Toh et al., 2017; Willis et al., 2018; Romanelli et al., 2019).

Here, we review the current knowledge of extracellular vesicles (EV) and MSCs-derived extracellular vesicles (MSCs-EV), and highlight the application studies of MSCs-EV for treatment of bone disorders by focusing on osteoarthritis (OA), rheumatoid arthritis (RA), osteoporosis (OP), and bone fracture. Furthermore, we discuss the key issues of MSCs-EV for clinical application.

## Extracellular Vesicles (EV)

### EV: Definition, Classification, and Function

Exosomes are a type of EV and the term “exosome” was first adopted by Trams et al. (1981) and refers to the EV formed by the endosomal system (Harding et al., 1983; Pan and Johnstone, 1983). Although the word “exosome” is commonly seen in literature, it is difficult to obtain purified exosomes, which make “exosome” ambiguous. Therefore, the International Society for Extracellular Vesicles (ISEV) has proposed minimal information for studies of extracellular vesicles 2018 (MISEV2018) in relation to the nomenclature of EV, which is defined for particles naturally released from the cell that are delimited by a lipid bilayer and cannot replicate (Thery et al., 2018). Since there is still lack of consensus on specific markers of EV subtypes, such as exosomes and ectosomes, the ISEV recommends the usage of EV, a collective term covering various subtypes of cell-released, membranous structures, called exosomes, microvesicles, microparticles, ectosomes, oncosomes, apoptotic bodies, and many other names (Thery et al., 2018). According to the uncertainty of what types of vesicles have been studied in many studies, in this review, we use the word “EV” and use the term as mentioned in the original work.

There is a different classification of EV proposed by different literature, ranging from two to six major different EV types (Cocucci et al., 2009; Thery et al., 2009; Beyer and Pisetsky, 2010; Mathivanan et al., 2010). However, due to the difficulty met in practice (e.g., the vesicles’ preparations are heterogeneous) and insufficient evidence for some EV types, van der Pol et al. (2012) have proposed four different types of eukaryotic cell-derived EV: (1) endosome-origin exosomes (50–100 nm); (2) plasma membrane-derived MV (20–1000 nm); (3) plasma membrane-derived membrane particles (50–600 nm); and (4) apoptotic vesicles (1000–5000 nm) from the plasma membrane and endoplasmic reticulum. However, at present, there is still no consensus on the classification of EV as stated by ISEV (Thery et al., 2018).

As particles naturally secreted from the cell, EV has been identified as vital mediators of paracrine communication (Batiz et al., 2015; Jing et al., 2018) and demonstrated a key role in different processes, such as angiogenesis (Qi et al., 2016), antigen presentation (Shenoda and Ajit, 2016), apoptosis (Qi et al., 2019), coagulation (Fricke et al., 2017), cellular homeostasis (Takahashi et al., 2017), inflammation (Zhang S. et al., 2019). EV is involved in numerous biological processes, including intercellular signaling, cell adhesion, waste management and protection against stress, coagulation, and vascular function and integrity, which has been summarized comprehensively by van der Pol et al. (2012).

### Biogenesis and Components of EV

**FIGURE 1:**
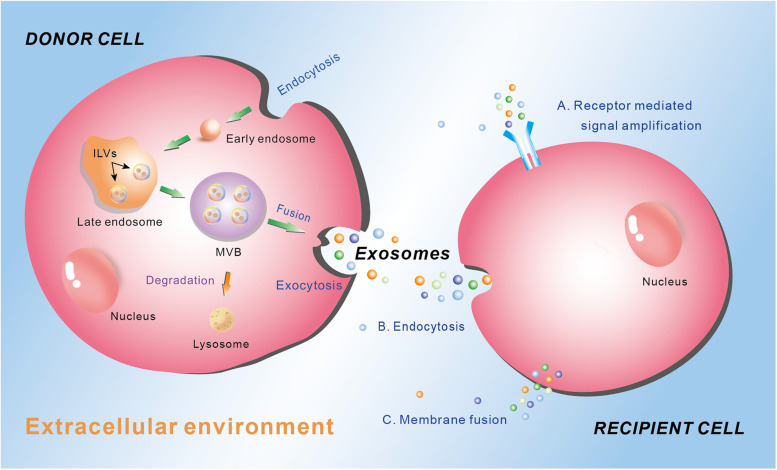
Schematic diagram of the biogenesis and secretion of exosomes, and their function ways to the recipient cell. Donor cell plasma membrane invaginate through endocytosis to form early endosome, which subsequently develop into late endosome via endosomal membrane budding inward to form intraluminal vesicles (ILVs). The late endosome constantly buds inward and form ILVs, which keep accumulating and constitute multivesicular body (MVB). MVB can either be degraded by lysosome or fuse with the plasma membrane to permit the ILVs to be released into the extracellular environment through exocytosis as exosomes. Exosomes mediate their effects on the recipient cells through three main manners: **(A)** receptor mediated signal amplification, **(B)** endocytosis, and **(C)** membrane fusion.

Different type of EV is generated through different way and contains different cargos, which depends on the cell types and the physiological conditions. The biogenesis of exosomes is well-studied, which involves the exocytosis of multivesicular endosomes (Figure 1). Generally, the biogenesis of exosome contains three phases: endocytosis, multivesicular body (MVB) development, and release (Figure 1) (Gurunathan et al., 2019). At the beginning, endocytic vesicles are produced from the plasma membrane (PM) to become early endosomes, which then develop into late endosomes via endosomal membrane budding inward to form intraluminal vesicles (ILVs). Subsequently, the late endosomes constantly bud inward and form ILVs, which keep accumulating and constitute MVBs. Finally, the MVBs may either be degraded by lysosome, or fuse with the plasma membrane to permit the ILVs to be released into the extracellular environment. The released vesicles are called exosomes (Figure 1) (van Niel et al., 2006; Dong et al., 2019). The exosomes play crucial roles in intercellular communication by regulating the recipient cell function. There are mainly three ways for exosomes regulating recipient cell function. First, exosomes interact with the membrane receptors of the recipient cell to activate the signaling cascade in the cell. Second, the endocytosis occurs in the recipient cell to ingurgitate the exosomes. Third, exosomes integrate own cargos with recipient cell membrane, then transfer the lipids, nucleic acids (mRNAs and miRNAs) and proteins through membrane fusion or by an endocytosis pathway, leading to numerous biological processes (Figure 1) (Yáñez-Mó et al., 2015).

At present, the well-known mechanism of exosome generation is driven by the endosomal sorting complex required for transport (ESCRT), which consists of approximately 30 proteins that form four complexes (ESCRT-0, ESCRT-I, ESCRT-II, ESCRT-III) with associated proteins including vacuolar protein sorting-associated protein 4 (VPS4), vesicle trafficking 1 (VTA-1), and apoptosis-linked gene 2 (ALG-2)-interacting protein X (Alix) (Hanson and Cashikar, 2012). They give the exosomes membrane a flexible state, which make exosomes be transported through the cytoplasm. Moreover, ESCRT-independent mechanism has been demonstrated for exosome biogenesis and secretion, in which way, although the four subunits of ESCRTs are silent, MVBs can still be generated (Trajkovic et al., 2008; Frydrychowicz et al., 2015). The review written by Colombo et al. (2014) provides a comprehensive understanding of the mechanism of exosome biogenesis.

**FIGURE 2:**
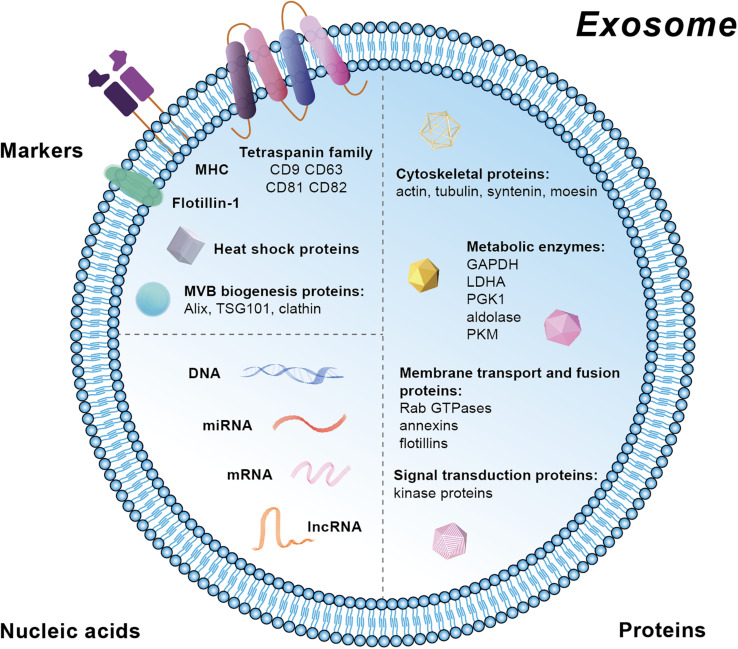
Schematic diagram of markers and the molecular composition of exosomes.

Extracellular vesicles contain markers from the parent cells and therefore their subtypes can be identified and characterized. Specific markers in exosomes are mainly located at the membrane, including the tetraspanin family (CD81, CD82, CD63, and CD9), major histocompatibility complex (MHC), and lipid raft such as Flotillin-1 (Figure 2). Moreover, heat shock proteins (HSP) and the proteins related with MVB biogenesis are also markers for exosomes (Figure 2). Besides these membrane molecules, there are other proteins and nucleic acids within the exosomes. The proteins are mainly related with MVBs biogenesis (Alix, TSG101, and clathrn), cytoskeleton (actin, tubulin, syntenin, and moesin), metabolism [GAPDH (Glyceraldehyde-3-phosphate dehydrogenase), LDHA (Lactic dehydrogenase A), PGK1 (Phosphoglycerate kinase 1), aldolase, and PKM (protein kinase)], membrane transport and fusion (Rab GTPases, annexins, and flotillins), and signal transduction (kinase proteins) (Chaput and Thery, 2011; Ohno et al., 2013; Raposo and Stoorvogel, 2013). The nucleic acids in exosomes are mainly mRNA, miRNA, long non-coding RNA (lncRNA), and DNA. Besides, various lipid compounds are found in exosomes, such as ceramide, sphingomyelin, phosphatidyl choline, phosphatidylserine, phosphatidyl ethanolamine, and cholesterol (Kowal et al., 2014) (Figure 2). Public on-line databases of EV composition are freely accessible at Exocarta^1^ and Vesiclepedia^2^.

### Isolation and Characterization of EV

**FIGURE 3:**
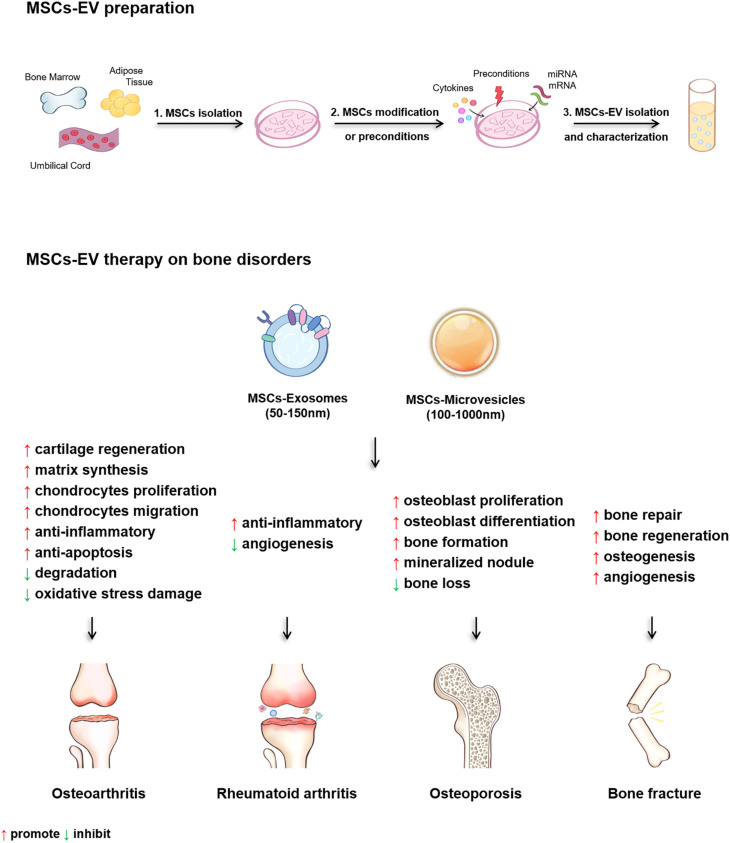
The diagram of the MSCs-EV preparation and the therapeutic effects of MSCs-EV on osteoarthritis (OA), rheumatoid arthritis (RA), osteoporosis (OP) and bone fracture.

Isolation of EV from specific tissues or cells is one important step toward further investigation and applications (Figure 3). Various techniques have been adopted to facilitate the isolation of EV based on different principles (Table 1). Traditional methods used for EV isolation are mainly based on EV properties (e.g., size and density), including ultracentrifugation, ultrafiltration, chromatography and precipitation. The ultracentrifugation process requires high centrifugal forces, up to 1,000,000 *g*. Ultracentrifugation is suitable for large sample volumes, but not for small volumes because it is time-consuming, labor-intensive and requires expensive equipment (Witwer et al., 2013). In addition, protein, aggregates, apoptotic bodies, and other non-exosomal particles may be present in EV obtained by ultracentrifugation, which is the major shortcoming of this method (Konoshenko et al., 2018). There are two types of ultracentrifugation: differential ultracentrifugation and density gradient ultracentrifugation. These two methods are used in order to increase the efficiency of particle separation and to obtain purer EV (Fernandez-Llama et al., 2010). Another isolation method is ultrafiltration, which merely depends on size or molecular weight. Ultrafiltration is rapid and does not require expensive devices, but it is difficult to remove contaminating proteins (Zeringer et al., 2015). Size-exclusion chromatography allows the separation of exosomes from proteins, but not from MV, protein aggregates, lipoparticles, macromolecules, or particulate matter (Gurunathan et al., 2019). Size-exclusion chromatography can be used in combination with ultracentrifugation for higher yields of EV (Lai et al., 2010). Precipitation isolates EV by capturing a certain size, and using simple, rapid, low-speed centrifugation on the bench top at 1,500 *g* in “polymer nets” (Gurunathan et al., 2019). Because it is easily operated and does not require specialized equipment, precipitation allows to be integrated into clinical usage and it can be applied for large sample sizes (Konoshenko et al., 2018). The disadvantage of this method is that there is no specificity for non-exosomal material, such as protein aggregates, which may be co-isolated with the exosomes resulting in low purity (Peterson et al., 2015). In addition, polymer-based precipitation is also used for EV isolation based on the changes in EV solubility and/or aggregation (Zeringer et al., 2015). *System Biosciences, LLC.* offers a proprietary reagent named ExoQuick, which can be used to purify exosomes from a wide variety of tissue culture media, and certain biofluids^3^. In order to isolate more specific EV populations, immunological methods are used based on highly specific interactions with the molecules (e.g., lipids, proteins, and polysaccharides) exposed on the EV surface. This approach is particularly useful when the protein expressed on the EV surface lacks a soluble counterpart (Gurunathan et al., 2019). Immuno-affinity is simple, rapid, and compatible with the laboratory equipment, while it is unstable and not suited for isolating EV from large quantities of biological samples (Konoshenko et al., 2018). Moreover, a new method utilizing aqueous two-phase system is adopted to isolate high-purity EV by preventing the protein contamination in the EV fraction (Kim et al., 2015). Recently, microfluidics-based technologies have become a trend for EV isolation, especially for microscale isolation, detection, and analysis of exosomes (Konoshenko et al., 2018; Gurunathan et al., 2019). Microfluidic devices utilizes the usual separation determinants and innovative sorting principles, mainly including: (a) trapping exosomes with an immune-affinity approach (microfluidic chip, “Exochip,” magnetic capture beads) (Chen C. et al., 2010; Kanwar et al., 2014; Shao et al., 2015); (b) membrane-based filtration (double filtration) (Liang et al., 2017); (c) trapping exosomes on porous structures (nanowire micropillars) (Wang et al., 2013); (d) acoustics (acoustic nano-filter system); (e) lateral displacement (nanoscale lateral displacement arrays) (Wunsch et al., 2016); and (f) viscoelastic flow (field-free microfluidic sorting) (Zhou J. et al., 2019).

**TABLE 1:** The isolation techniques of EV and their advantages and disadvantages.

**Isolation techniques**	**Principle**	**Advantages**	**Disadvantages**	**References**
Differential ultracentrifugation	Size	Conventionality, isolation from large sample volumes	Inapplicability for small sample, time-consuming, labor cost, expensive equipment-dependent	Livshits et al., 2015
Density gradient centrifugation	Density	Conventionality, isolation from large sample volumes, high purity	Inapplicable for small sample, time-consuming, labor cost, expensive instrument-dependent	Greening et al., 2015
Ultrafiltration	Size or weight	Rapidness, high purity, no limitations on sample volume	Aggressive, difficult to remove contaminating proteins	Lobb et al., 2015
Size exclusion chromatography	Size	High purity, removal of soluble proteins, preserve vesicle integrity, high sensitivity	Low yield, need of further concentration steps, limitations on sample volume and number of separated peaks	Gamez-Valero et al., 2016
Precipitation	Size	Facility, rapidness, reservation of EV integrity, no need in additional equipment, high ion concentrations	Low purity, protein contaminations	Crossland et al., 2016
Polymer precipitation	Solubility or aggregation	Preservation of EV integrity, no additional equipment, pH close to physiological range, high ion concentration	Contamination and retention of the polymer	Wang and Sun, 2014
Immuno-affinity	Membrane surface proteins	Easy, rapid, High purity, higher capture efficiency, no volume limitations	Unstable, inapplicability for large sample	Konoshenko et al., 2018
Microfluidics techniques	Membrane surface targeted markers, size, acoustics, viscoelastic flow	Rapidness, consume low volumes of sample and reagents, high purity, efficiency	High-cost, expensive equipment-dependent	Contreras-Naranjo et al., 2017

Characterization of EV is essential for determining their biochemical properties and biological functions. The characterization mainly includes the evaluation of the size, structure, surface biochemical markers, concentration and contents of EV. There are numerous approaches for characterizing different biochemical properties of EV. To detect EV biochemical markers, western blotting, flow cytometry, microfluidic-based technique and nanoparticle-tracking analyses (NTA) are normally used. EV components, including proteins, mRNA, miRNA, and lipid subsets can be detected by conventional test method such as western blotting, PCR and flow cytometry. As technology advances, high throughput transcriptomic studies have identified a wide range of mRNA and miRNA data sets based on microarray and next-generation sequencing (NGS) analyses leading to a comprehensive data classification. For detecting size and structure of EV, NTA, Tunable-resistive pulse sensing (TRPS), Dynamic-light scattering (DLS), Photon-correlation spectroscopy (PCS), Atomic-force microscopy (AFM), Transmission electron microscopy (TEM), Raman spectroscopy and Flow cytometry are used. Meanwhile, NTA and TRPS can be used to detect concentration.

## Mesenchymal Stem Cells (MSCs) and MSCs-Derived EV (MSCs-EV)

### Mesenchymal Stem Cells (MSCs)

MSCs, also known as mesenchymal stromal cells (MSCs), are multipotent fibroblast-like cells with the potential for self-renewal and multilineage differentiation (Hu et al., 2018). The Mesenchymal and Tissue Stem Cell Committee of the International Society for Cellular Therapy (ISCT) has proposed the minimal criteria to define human MSCs (Dominici et al., 2006). First, MSCs must be plastic-adherent when maintained in standard culture conditions. Second, MSCs must express CD105, CD73, and CD90, and lack expression of CD45, CD34, CD14 or CD11b, CD79 alpha or CD19 and HLA-DR surface molecules. Third, MSCs must differentiate to osteoblasts, adipocytes and chondroblasts *in vitro*.

MSCs reside in almost all post-natal organs and tissues such as bone marrow, adipose, liver, lung, spleen, and muscle (da Silva Meirelles et al., 2006). MSCs have the ability to proliferate extensively and maintain the ability to differentiate into multiple cell types (e.g., osteoblasts, adipocytes, chondrocytes, and fibroblasts), which endow MSCs with stem cell nature (Pittenger et al., 1999). Besides, MSCs can migrate to the sites of damage and secrete anti-inflammatory and growth factors to maintain cellular homeostasis and promote damage repair.

With the above characteristics, MSCs are commonly used in cell therapy for regenerative medicine and immunotherapy (Squillaro et al., 2016; Galipeau and Sensebe, 2018). MSCT has caused increasing attention and is regarded as a potential new therapy for a variety of human diseases associated with brain, liver, kidney, bone, and so on (Hu et al., 2018; Chakari-Khiavi et al., 2019; Hu et al., 2019; Missoum, 2020). However, the direct use of MSCs for treating diseases encounters some problems. For example, MSCs inoculated into damage site show low survival rate and loss of function after 1 week (Pak et al., 2003; Breitbach et al., 2007; Rani et al., 2015). Besides, intraarterial MSCs administration may lead to occlusion in the distal vasculature due to their relatively large cell size (Furlani et al., 2009). In addition, MSCs may genetically unstable and undergo chromosomal abnormalities, even develop malignant tumor or generated calcification (Yoon et al., 2004; Tolar et al., 2007; Jeong et al., 2011). These issues may lower its security for treating human disease.

Recently, numerous studies demonstrate the therapeutic effect of MSCs is primarily mediated by the paracrine secretion of cytokines, growth factors and EV (Wang et al., 2015; Zhou Y. et al., 2019). MSCs-EV are identified as key mediators for the paracrine effects and gradually become the focus in regenerative medicine and disease treatment (Yang et al., 2017; Chang et al., 2018).

### MSCs-EV

Over the years, of a wide array of bioactive molecules, MSCs secretions such as growth factors, cytokines and chemokines have been proved to play vital biological roles. Among these secretory products, none of them, by itself, can fully decipher the mechanism of MSCs (Meirelles Lda et al., 2009). In 2009, MSCs-derived MV were found to alleviate glycerol-induced acute tubular injury. The size of these MV ranges from 80 nm to 1 μm, with a mean value of 135 nm (Bruno et al., 2009). Subsequently, Lai et al. isolated and characterized MSCs-derived exosome for the first time from conditioned medium of human embryonic-derived MSCs (hESC-MSCs) (Lai et al., 2010). These membrane vesicles feature a diameter of 50–100 nm with an endosomal origin, housing abundant exosome-associated proteins including Alix, TSG101 and tetraspanins (Lai et al., 2010). MSCs-exosomes have special membrane binding proteins such as CD73, CD44, and CD29 compared with other EV (Lai et al., 2012). To date, more than 850 unique gene products and 150 miRNAs have been identified in MSCs-exosomes (Chen T.S. et al., 2010; Lai et al., 2012). In addition, MSCs have been demonstrated as the most prolific producer of mass exosomes (Yeo et al., 2013). They can produce large numbers of exosomes and generate permanent cell lines through cell immortalization, whose yield is not affected in quantity or quality, thus ensuring a sustainable and reproducible production of exosomes from MSCs (Yeo et al., 2013).

Previous studies have shown that the function of MSCs-EV may vary depending on the source of the MSCs, thus affecting the biological effects of MSCs-EV (Baglio et al., 2015; Del Fattore et al., 2015; Lopez-Verrilli et al., 2016; Borger et al., 2017). Thus, MSCs-EV derived from different MSCs may show different clinical efficacy. It is important to determine the optimal source of MSCs. In addition, the environment of MSCs can change the content of EV derived from MSCs, thus affecting and changing the tissue environment. MSCs-EV may have the versatility and capacity to interact with multiple cell types within the immediate vicinity and remote areas to elicit appropriate cellular responses to keep the maintenance of a dynamic and homeostatic tissue microenvironment (Lai et al., 2015).

## MSCs-EV and Osteoarthritis (OA)

Osteoarthritis is the most common chronic joint disease around the world, with an incidence of 10–20% in the population over 50 years old. As the population ages and obesity increases, the incidence of OA is expected to double within the next 30 years (Blanco et al., 2011). It has become the fastest growing major health concern worldwide. OA is characterized by the degradation of the articular cartilage, the degeneration of menisci and ligaments, the thickening of the subchondral bone, and the formation of osteophytes (Loeser et al., 2012). The pathogenesis of OA is complex, involving multiple factors (e.g., age, body mass index, and genetics), multiple tissues, and processes.

There is still currently no effective treatment for OA. Most treatments are applied to manage pain, stiffness and swelling to improve joint mobility, and joint replacement is the only option for treating the entire joint dysfunction. Although the current physiotherapy or pharmacological therapy can temporarily relieve the clinical symptoms, it is difficult to fully restore joint function and also has a high risk of instability and infection (Keith, 2012; Stiehler et al., 2015; Xin-Wei et al., 2015).

The potential of MSCs to treat OA has been extensively studied (Qi et al., 2012; Vega et al., 2015; Davatchi et al., 2016). Recent studies have discovered that MSCs function in a paracrine manner by secreting cytokines, growth factors and EV (D’souza et al., 2015; Giebel et al., 2017; Fan et al., 2020) and exosomes derived from MSCs have shown the capability to protect cartilage and bone from degradation in OA through reinducing the expression of chondrocyte markers (type II collagen, aggrecan), inhibiting catabolic (MMP13 (matrix metalloproteinase 13), ADAMTS5) and inflammatory markers, inhibiting macrophage activation, and protecting chondrocytes from apoptosis, which show anti-inflammatory and cartilage protection effects (Cosenza et al., 2017).

### Anti-inflammatory Effect of MSCs-EV in OA

Inflammation is an important factor in the onset and progression of OA (Nold et al., 2015; Burrello et al., 2016). In OA patients, activated macrophages and other innate immune cells release inflammatory cytokines and promote cartilage damage (Liu-Bryan, 2015). Infiltrations of B lymphocyte, T lymphocyte, plasma cells, T-helper cells, and Human Leukocyte Antigen-antigen D Related (HLA-DR)-positive dendritic-like cell were observed in the inflamed synovium (Lindblad and Hedfors, 1987). Moreover, catabolic factors [e.g., interleukin-1α (IL-1α), tumor necrosis factor-α (TNF-α)] are present in OA joints and inhibit the chondrogenesis of stem cells (Heldens et al., 2012).

Recent evidence suggest that adipose tissue-derived mesenchymal stem cells (AD-MSCs)-exosomes (size of 104 ± 19 nm) and MV (size of 279 ± 94 nm) show great potential in anti-inflammatory and preventing degeneration processes in OA (Tofiño-Vian et al., 2018). Exosomes (∼120 nm) and MV/microparticles (>150 nm) derived from bone marrow mesenchymal stem cells (BM-MSCs) show anti-inflammatory function through inhibiting T lymphocyte proliferation, stimulating macrophage polarization toward anti-inflammatory phenotype, and decreasing the percentage of CD4^+^ and CD8^+^ T cell subsets (Cosenza et al., 2018). In addition, BM-MSCs-exosomes (30–250 nm) induce conversion of T helper type 1 (Th1) cells into T helper type 2 (Th2) cells and reduce the potential of T cells to differentiate into interleukin 17-producing effector T cells (Th17) (Chen et al., 2016). These findings demonstrate that MSCs-EV suppress pro-inflammatory response by reducing inflammation and promoting anti-inflammatory responses that maintain the immune balance. Furthermore, in the rat model of temporomandibular joint osteoarthritis (TMJ-OA), hESC-MSCs-exosomes (size of 100–200 nm, density of 1.10–1.19 g/ml) can relieve pain and repair osteoarthritic TMJ. MSCs-exosomes increase a well-coordinated response of attenuating inflammation, enhance matrix synthesis, while reduce OA joint cellular apoptosis and matrix degradation to achieve overall joint homeostasis, which finally promote TMJ repair and regeneration (Zhang S. et al., 2019). MSCs-exosomes function through adenosine receptor-mediated adenosine activation of protein-serine-threonine kinase (AKT), extracellular regulated protein kinases (ERK) and Adenosine 5′-monophosphate (AMP)-activated protein kinase (AMPK) phosphorylation to reduce inflammation and restore matrix homeostasis in OA. These therapeutic effects were achieved by enhancing sulfated glycosaminoglycan (s-GAG) matrix synthesis impeded by interleukin-1β (IL-1β), while suppressing IL-1β-induced nitric oxide and MMP13 production (Zhang S. et al., 2019). Cosenza et al. (2017) also found that exosomes (112 ± 6.6 nm) and microparticles (223 ± 15.6 nm) from murine BM-MSCs exerted similar functional effect *in vitro* by re-establishing chondrocyte homeostatic state, protecting chondrocytes from apoptosis and stimulating macrophage polarization toward anti-inflammatory phenotype. Therefore, MSCs-EV possess the immunomodulatory properties and accelerate the recovery of cartilage and joint in OA.

### Cartilage Protection and Regeneration Effect of MSCs-EV in OA

The main pathology of early stage OA is the degeneration of chondrocytes, resulting in damage to articular cartilage. Metabolic and structural changes in articular cartilage play a major role in the initiation and progression of OA. MSCs-EV exert important therapeutic effect on OA by protecting cartilage from degradation and promoting cartilage regeneration, which is now the focus of clinical therapy.

The efficacy of hESC-MSCs-exosomes (a modal size of 100 nm) on cartilage repair was firstly reported in 2016 (Zhang S. et al., 2016). After treatment with exosomes, the rat model of osteochondral defect displayed almost complete neotissue coverage with good surface regularity and complete integration with the surrounding cartilage. hESC-MSCs-exosomes accelerated neotissue filling and enhanced matrix synthesis of type II collagen and s-GAG, demonstrating the capacity of MSCs-exosomes in cartilage repair and regeneration (Zhang S. et al., 2016). It has also been demonstrated that MSCs-EV protect chondrocytes from apoptosis, balance the anabolic and catabolic processes and re-establish chondrocyte homeostatic state via balancing the synthesis and degradation of cartilage matrix, thus protect cartilage and bone from degradation (Cosenza et al., 2017; Wang et al., 2017; Wu et al., 2019). All these observations demonstrate the therapeutic effects of MSCs-EV on OA by re-establishing cartilage homeostasis, protecting and regenerating cartilage.

Several key cargo components of MSCs-EV have shown important roles in mediating the therapeutic effects of MSCs-EV. MiRNAs, a large cargo of MSCs-EV (Chen T.S. et al., 2010), are identified as key regulators in mediating the therapeutic roles of MSCs-exosomes on OA by targeting different molecules or signaling pathways (Toh et al., 2017). AD-MSCs-EV (ranged in size from 40–50 nm to 300–400 nm) have been demonstrated to affect fibroblast-like synoviocytes (FLS) behavior in a model of chronic inflammation through direct interaction with hyaluronan matrix and miRNA release (Ragni et al., 2019). They reduced the expression of pro-inflammatory cytokines and chemokines in a chronic model of FLS inflammation. Through bioinformatics analysis, EV-embedded miRNAs regulate the main pathways, which is strictly associated with synovial inflammation in OA (Ragni et al., 2019). Further research revealed AD-MSCs-EV (50–400 nm in diameter)-embedded miRNAs alter cartilage homeostasis and macrophage polarization, supporting the protective and pro-regenerative effects in joint (Ragni et al., 2020). miR-92a-3p derived from BM-MSCs-exosomes (50–150 nm) regulates cartilage development and homeostasis through targeting Wnt5a in both chondrogenesis and OA pathogenesis as a negative regulator (Mao et al., 2018). miR-135b derived from hESC-MSCs-exosomes assists transforming growth factor β1 (TGF-β1) to promote chondrocyte proliferation by down-regulating Specificity Protein 1 (Sp1), thus promotes cartilage repair in OA (Wang et al., 2018). Infrapatellar fat pad (IPFP)-derived mesenchymal stem cells (IPFP-MSCs)-exosomes (average size of 121.9 nm) significantly enhance the autophagy level of chondrocytes via miR100-5p-mediated inhibition of mammalian target of rapamycin (mTOR) signaling pathway (Wu et al., 2019). Exosomes (30–150 nm) derived from miR-140-5p overexpressing synovial MSCs (SMSCs) activate yes-associated protein (YAP), decrease extracellular matrix (ECM) secretion, and promote proliferation and migration of articular chondrocytes via Wnt5a and Wnt5b, which in turn ameliorate OA (Tao et al., 2017). These studies demonstrate that MSCs-EV attenuate OA progression through the delivery of miRNAs, which exhibit as potential targets for future therapy (Table 2).

**TABLE 2:** MSCs-EV cargo involved in regulation of chondrogenesis and cartilage homeostasis.

**MSCs-EV cargo**	**Target**	**Effects and mechanism**	**References**
miR-92a-3p	Wnt5a	Regulate cartilage development and homeostasis by targeting Wnt5a	Mao et al., 2018
miR-135b	Sp1^a^	Promote chondrocyte proliferation and cartilage repair in OA^b^ by down-regulating Sp1^a^ in chondrocyte thus affect TGF-β1^c^	Wang et al., 2018
miR-100-5p	mTOR^d^	Inhibit mTOR signaling pathway to enhance the autophagy level of chondrocytes	Wu et al., 2019
miR-140-5p	YAP^e^	Decrease ECM^f^ secretion and induce proliferation and migration of articular chondrocytes via activating YAP, Wnt5a and Wnt5b	Tao et al., 2017
lnc KLF3-AS1	IL-1β^g^	Inhibit IL-1β-induced apoptosis of chondrocytes and promote cartilage repair in a rat model of OA through decreasing Runx2^h^ and MMP13^i^ expression while increasing Col2α1^j^ and aggrecan expression	Liu et al., 2018
CD73	AKT, ERK	Osteochondral defects repair, increased cellular proliferation and infiltration, enhanced matrix synthesis and a regenerative immune phenotype, attributing to exosomal CD73-mediated adenosine activation of AKT and ERK signaling	Zhang et al., 2018

*^*a*^Specificity protein 1; ^*b*^Osteoarthritis; ^*c*^Transforming growth factor β1; ^*d*^Mammalian target of rapamycin; ^*e*^Yes-associated protein; ^*f*^Extracellular matrix; ^*g*^Interleukin-1β; ^*h*^Runt-related transcription factor 2; ^*i*^Matrix metalloproteinase 13; ^*j*^Collagen, type II, alpha 1.*

In addition to miRNA, a few lncRNAs have been found as main components of EV and to function as novel biomarkers and therapeutic targets for various diseases (Naderi-Meshkin et al., 2019). Exosomal lncRNA KLF3-AS1 derived from human MSCs (hMSCs) has been demonstrated to inhibit IL-1β induced apoptosis of chondrocytes and promote cartilage repair in a OA rat model by decreasing Runt-related transcription factor 2 (Runx2) and MMP13 expression while increasing collagen, type II, alpha 1 (Col2a1) and aggrecan expression (Liu et al., 2018). This suggests the important role of lncRNA in mediating the therapeutic effect of MSCs-exosomes on OA, demonstrating a new possible mechanism. In addition, protein components in MSCs-exosomes have been suggested to be linked with OA recovery. Zhang et al. (2018) demonstrated the therapeutic effect of vesicular CD73 in cartilage repair and regeneration (Zhang et al., 2018). hESC-MSCs-Exosomes (a modal size of 100 nm, density of 1.10–1.19 g/ml) mediated repair of osteochondral defects, which was characterized by increased cellular proliferation and infiltration, enhanced matrix synthesis and a regenerative immune phenotype. These could attribute to exosomal CD73-mediated adenosine AKT and ERK signaling (Zhang et al., 2018) (Table 2).

As mitochondrial dysfunction is one important cause of OA (Blanco et al., 2011) and MSCs-EV are important for intercellular mitochondria communication (Cao et al., 2020), suggesting the possibility of MSCs-EV in treating OA by regulating mitochondria function. Mitochondrial dysfunction and oxidative stress damage are associated with apoptosis, senescence and various pathological conditions, which are hallmarks of OA. In OA, chondrocytes lose their metabolic flexibility, decrease their cellular mitochondrial biogenesis, and increase their mitochondrial DNA (mtDNA) damage (Chen et al., 2019). Qi et al. (2019) have found that BM-MSCs-exosomes (50–150 nm) suppress IL-1β-induced chondrocyte apoptosis, which was partly due to mitochondrial dysfunction, through inhibiting the phosphorylation of p38 and ERK1/2, and stimulating the phosphorylation of AKT signaling pathway. Moreover, Chen et al. (2019) have reported that BM-MSCs-exosomes (40–110 nm) restore mitochondrial function and oxidative stress damage in degenerative cartilage and balance the energy metabolism, which promote cartilage regeneration. Based on these findings, they furtherly fabricated a 3D printed cartilage extracellular matrix/gelatin methacrylate/exosome (ECM/GelMA/exosome) scaffold with radially oriented channels and found it significantly facilitated the cartilage regeneration in the animal model (Chen et al., 2019).

## MSCs-EV and Rheumatoid Arthritis (RA)

Arthritis is a chronic autoimmune disease involving joints. It is characterized by persistent inflammation of joints. In RA, auto-reactive T cells and B cells are activated because of defective immune regulation. These cells proliferate and differentiate into pathological T cells and plasma B cells, which produce auto-reactive antibodies, eventually leading to inflammation and degradation of joints (Pozsgay et al., 2017). As mentioned in OA section that MSCs-EV exert anti-inflammatory effects, they have also exerted an immunomodulatory function in RA and show therapeutic effects on joint destruction (Chen et al., 2018; Cosenza et al., 2018).

Collagen-induced arthritis (CIA) is one of the most accepted animal models of RA for its capacity to simulate RA inflammatory symptoms clinically and biologically. Cosenza et al. (2018) have demonstrated that Exosomes (∼120 nm) and MV/microparticles (>150 nm) derived from BM-MSCs exert an indirect inhibitory effect on T lymphocyte and B lymphocyte proliferation in CIA murine model, providing the first evidence that MSCs-exosomes exert an immunomodulatory function in RA. Chen et al. (2018) have also found that BM-MSCs-exosomes show therapeutic effects on joint destruction in RA, partly due to the expression of miR-150-5p in exosomes. After being treated with MSCs-derived miR-150-5p exosomes (Exo-150), the expression of the RA markers including matrix metalloproteinase 14 (MMP14) and vascular endothelial growth factor (VEGF) in CIA mice was down-regulated. Exo-150 inhibited migration of FLS from RA, and angiogenesis *in vitro*, and alleviated arthritis in CIA mice *in vivo*. These findings indicate that EV facilitate the direct intracellular transfer of miRNAs and represent a potential therapeutic strategy for RA (Chen et al., 2018).

At present, only a few studies have reported the therapeutic effect of MSCs-EV on RA and the mechanism is unclear. As MSCs-EV play roles in immunoregulation and cartilage protection and regeneration, this may partially contribute to the therapeutic effect of MSCs-EV on RA. More research is necessary to prove the effects of MSCs-EV in treating RA.

## MSCs-EV and Osteoporosis (OP)

Osteoporosis is an age-related systemic bone disease characterized by reduced bone mass, destroyed bone microstructure, weakened bone strength, which causes increased bone brittleness and fracture risk. As one major disease worldwide, osteoporosis seriously affects people’s health and increases society’s financial burden. Therefore, efficient therapy of osteoporosis is one main concern of the world.

MSCs-EV have been indicated as a novel therapeutic method for osteoporosis. Qi et al. (2016) have found that exosomes (50–150 nm) secreted by human-induced pluripotent stem cell-derived mesenchymal stem cells (hiPSC-MSCs) effectively promote osteoblast proliferation, differentiation and bone formation, thus improve bone regeneration in osteoporotic rats. Zuo et al. (2019) have reported that exosomes (40–100 nm) derived from BM-MSCs alleviate radiation-induced bone loss in a rat model. After irradiation to rats, BM-MSCs-exosomes were intravenously injected into the rats immediately. The results showed that BM-MSCs-exosomes reduced intracellular reactive oxygen species (ROS) to protect cells from damage, accelerated DNA repair in the recipient BM-MSCs and partially rescued cell proliferation. Moreover, BM-MSCs-exosomes decreased the senescence-associated protein expression and restored the differentiation potential of irradiated BM-MSCs (Zuo et al., 2019). Meanwhile, BM-MSCs-exosomes reduced adipogenic gene expression and increased osteogenic gene expression of recipient BM-MSCs (Zuo et al., 2019). These results demonstrate that MSCs-EV promote bone regeneration via reducing ROS, accelerating DNA repair, restoring cell (especially osteoblast) proliferation and differentiation potential, decreasing the senescence-associated protein and adipogenic gene expression and increasing osteogenic gene expression, thus alleviate osteoporosis.

Present studies demonstrate that MSCs-EV alleviate osteoporosis by activating several signaling pathways. Wnt/β-catenin signaling is a key pathway for bone development and homeostasis (Baron and Kneissel, 2013; Zhong et al., 2014). The activation of Wnt/β-catenin signaling promotes preosteoblast to differentiate into osteoblasts. Zuo et al. (2019) have found that BM-MSCs-exosomes promotes osteogenesis, reduced the decrease in bone mass induced by irradiation by activating Wnt/β-catenin pathway in the recipient BM-MSCs. Zhao et al. (2018) found that BM-MSCs-exosomes (about 40 nm) alleviated the osteoporosis progression by promoting the osteoblast proliferation through mitogen-activated protein kinase (MAPK) signaling pathway. Moreover, Liu et al. have demonstrated the therapeutic function of BM-MSCs-exosomes on osteopenia in Fas-deficient-MRL/lpr mice (Liu et al., 2015). The Fas-deficient-MRL/lpr mice exhibit Fas-deficiency that causes elevation of intracellular miR-29b levels and downregulation of DNA methyltransferase 1 (Dnmt1) in BM-MSCs, which causes hypomethylation of the Notch1 promoter and activation of Notch signaling, thus results in impaired osteogenic differentiation. MSCs-exosomes reduce intracellular miR-29b levels by transfer Fas to the recipient MRL/lpr BM-MSCs and recover Dnmt1-mediated Notch1 promoter hypomethylation, elevate mineralized nodule formation, expression of Runx2 and alkaline phosphatase (ALP) of MRL/lpr BM-MSCs, thus increase bone formation, trabecular bone volume, bone mineral density (BMD), bone volume/total volume (BV/TV), mineral apposition rate (MAR) and bone formation rate per bone Surface (BFR/BS) in Fas-deficient-MRL/lpr mice (Liu et al., 2015). This study demonstrates that BM-MSCs-exosomes function through Fas/miR-29b/Dnmt1/Notch epigenetic cascade to regulate the recipient cell function.

## MSCs-EV and Bone Fracture

Besides the critical role in alleviating osteoporosis, MSCs-EV show key roles in fracture healing. In order to ensure bone fracture healing, two basic conditions must be met: good blood transport and stable fixation. hMSCs-exosomes (80–100 mm) have been found to promote bone regeneration by enhancing angiogenesis. MSCs-exosomes increased hMSC migration, induced mineral deposition and enhanced the differentiation potential of hMSCs into osteoblasts. In addition, MSCs-exosomes enhanced the expression of osteogenesis-related and angiogenesis-related genes, such as *COL I, ALP*, and *VEGF*, and promoted bone formation *in vivo* (Takeuchi et al., 2019). The CD9^–/–^ mouse, which is an established strain with low-exosome productivity, shows a significant delay in endochondral ossification and fracture healing compared with the wild-type mouse. However, injection of BM-MSCs-exosomes (approximately 80 nm) rescues the fracture healing speed in CD9^–/–^ mice and increases that in the wild-type mice, indicating the potential therapeutic role of MSCs-exosomes in fracture healing (Furuta et al., 2016). In addition, MSCs-exosomes contain bone repair related cytokines such as monocyte chemotactic protein 1 (MCP-1), monocyte chemotactic protein 3 (MCP-3), stromal cell-derived factor-1 (SDF-1) and angiogenic factors, which accelerate fracture healing. Meanwhile, miRNAs derived from MSCs-exosomes are key regulators for fracture healing. miR-21 accelerates osteogenic bone formation, osteogenesis and angiogenesis, which promote fracture healing (Furuta et al., 2016). Furthermore, Zhang et al. have also demonstrated the therapeutic effect of MSCs-exosomes (approximately 100 nm) on bone fracture healing (Zhang Y. et al., 2019). They delivered umbilical cord mesenchymal stem cells (uMSCs)-exosomes carried by a HyStem-HP hydrogel to the fracture site in a rat model of femoral fracture and found that 14 days treatment significantly increased callus volumes, BMD, BV and BV/TV (Zhang Y. et al., 2019). In a separate study, hiPSC-MSCs-exosomes enhance the osteoinductivity of β-tricalcium phosphate (β-TCP) through activating the phosphatidylinositol-3-kinases/protein-serine-threonine kinase (PI3K/AKT) signaling pathway in hBMSCs. Based on the above findings, they designed hiPSC-MSCs-Exos functionalized β-TCP scaffold can effectively promote bone repair and regeneration in a rat model of calvarial bone defects (Zhang J. Y. et al., 2016). In a latest study, hypoxic uMSCs-exosomes (50–150 nm) promote fracture healing by transfering miR-126 (Liu et al., 2020). Compared with regular exosomes, hypo-exos function better in promoting angiogenesis, proliferation and migration of endothelial cells to a greater extent. Moreover, hypoxia preconditioning mediated enhanced production of exosomal miR-126 through the activation of hypoxia inducible factor 1 (HIF-1α). Hypoxia preconditioning represents a promising method for optimizing the therapeutic actions of MSC- exosomes in bone fracture healing (Liu et al., 2020). These findings verify the vital regulatory role of MSCs-EV in bone fracture healing, identifying it as a potential therapeutic method for bone fracture.

In summary, present studies demonstrate that MSCs-EV are effective for treating OA, RA, OP, and bone fracture both *in vitro* and in animal models (Table 3 and Figure 3). Several regulatory pathways have been demonstrated to function in MSCs-EV treating OA, RA, OP, and bone fracture, including AKT, ERK, AMPK, mTOR-autophagy, Wnt/β-catenin, MAPK, and Notch signaling pathways. For the treatment of OA, MSCs-EV reduce inflammation by activating adenosine of AKT and ERK, and the phosphorylation of AMPK. Moreover, MSCs-EV significantly enhance the autophagy level of chondrocytes through inhibiting mTOR signaling pathway. In terms of cartilage protection and regeneration, MSCs-EV activate YAP, decrease ECM secretion, and promote articular chondrocytes proliferation and migration via Wnt5a and Wnt5b. In addition, MSCs-EV suppress IL-1β-induced chondrocyte apoptosis, and enhance s-GAG matrix synthesis through inhibiting the phosphorylation of p38 and ERK1/2 thus restore matrix homeostasis and ameliorate OA (Table 3 and Figure 3). In the treatment of bone loss and fracture healing, MSCs-EV promote osteogenesis, reduced the decrease in bone mass by activating Wnt/β-catenin pathway, and alleviate the osteoporosis progression by promoting the osteoblast proliferation through MAPK signaling pathway. Furthermore, MSCs-EV recover Dnmt1-mediated Notch1 promoter hypomethylation, enhance mineralized nodule formation, elevate the expression of Runx2 and ALP, thus increase bone formation (Table 3 and Figure 3). These findings suggest potential clinical application of MSCs-EV in the treatment of bone disorders and also reveal the possible regulatory pathways that can be targeted for the clinical translation of MSCs-EV in treating bone disorders (Table 3 and Figure 3).

**TABLE 3:** Preclinical studies on the application of MSCs-EV for treating bone disorders.

**Origin of EV**	**Disease**	**Outcomes**	**Function way and mechanism**	**References**
BM-MSCs^a^	OA^b^	Attenuate inflammation, promote cartilage repair and regeneration	Inhibit T lymphocyte proliferation, stimulate macrophage polarization toward anti-inflammatory phenotype, restore mitochondrial function and oxidative stress damage, balance the energy metabolism, suppress mitochondrial dysfunction apoptosis of chondrocytes through inhibiting the phosphorylation of p38 and ERK1/2^c^, and stimulating of the phosphorylation of AKT^d^ signaling pathway, which promote cartilage regeneration	Chen et al., 2016, 2019; Cosenza et al., 2017, 2018; Mao et al., 2018; Qi et al., 2019
AD-MSCs^e^	OA^b^	Attenuate inflammation and modulate chondrocyte metabolism	Counteract the effects of IL-1β^f^	Tofiño-Vian et al., 2018
hESC-MSCs^g^	OA^b^	Attenuate inflammation, promote cartilage repair and regeneration	Reduce inflammation through adenosine receptor-mediated AKT^d^, ERK^c^ and AMPK^h^ phosphorylation, restore matrix homeostasis by enhancing matrix synthesis of type II collagen and s-GAG^i^ matrix synthesis	Zhang S. et al., 2016; Wang et al., 2017; Zhang et al., 2018; Zhang S. et al., 2019
IPFP-MSCs^j^	OA^b^	Protect cartilage and bone from degradation and maintain cartilage homeostasis	Related to miR100-5p-regulated inhibition of mTOR^k^-autophagy pathway	Wu et al., 2019
SMSCs^l^	OA^b^	Induces chondrogenic differentiation	Activate YAP^m^, decrease ECM^n^ secretion, and promote proliferation and migration of articular chondrocytes via Wnt5a and Wnt5b	Tao et al., 2017
BM-MSCs^a^	RA^o^	Attenuate RA inflammatory symptoms	Inhibit T lymphocyte and B lymphocyte proliferation, migration and invasion, inhibit angiogenesis via downregulating MMP14^p^ and VEGF^q^	Chen et al., 2018; Cosenza et al., 2018
hiPSC-MSC^r^	OP^s^	Improve osteoporosis, promote osteoblast proliferation, differentiation and bone formation	Up-regulate mRNA and protein expression of osteoblast-related genes	Qi et al., 2016
BM-MSCs^a^	OP^s^	Alleviate osteoporosis progression and bone loss, elevate mineralized nodule and bone formation	Accelerate DNA repair, reduce adipogenic gene expression, increase osteogenic gene expression, activating Wnt/β-catenin and MAPK^t^ signaling pathway, upregulate expression of Dnmt1^u^, Runx2^v^ and ALP^w^, downregulate expression of Notch1 and NICD	Liu et al., 2015; Zhao et al., 2018; Zuo et al., 2019
BM-MSCs^a^	Bone fracture	Promote fracture healing	Accelerate bone repair via cytokines, miRNAs such as miR-21, miR-4532, miR-125b-5p, and miR-338-3p in exosomes, promote osteogenic bone formation, osteogenesis and angiogenesis	Furuta et al., 2016; Takeuchi et al., 2019
uMSCs^x^	Bone fracture	Significantly increased callus volumes, BMD^y^, BV^z^ and BV/TV^aa^ and enhance fracture healing, promote angiogenesis, proliferation and migration	Through HIF-1α-mediated promotion of angiogenesis and transfer of miR-126	Zhang Y. et al., 2019; Liu et al., 2020
hiPSC-MSCs^r^	Bone fracture	Promote bone repair and regeneration	Activates the PI3K/AKT^ab^ signaling pathway	Zhang J. Y. et al., 2016

*^*a*^Bone marrow mesenchymal stem cells; ^*b*^Osteoarthritis; ^*c*^Extracellular regulated protein kinases; ^*d*^Phosphorylation of protein-serine-threonine kinase; ^*e*^Adipose tissue-derived mesenchymal stem cells; ^*f*^Interleukin-1β; ^*g*^Human embryonic-derived mesenchymal stem cells; ^*h*^Adenosine 5′-monophosphate (AMP)-activated protein kinase; ^*i*^Sulfated glycosaminoglycan; ^*j*^Infrapatellar fat pad (IPFP)-derived mesenchymal stem cells; ^*k*^Mammalian target of rapamycin; ^*l*^Synovial mesenchymal stem cells; ^*m*^Yes-associated protein; ^*n*^Extracellular matrix; ^*o*^Rheumatoid arthritis; ^*p*^Matrix metalloproteinase 14; ^*q*^Vascular endothelial growth factor; ^*r*^Human-induced pluripotent mesenchymal stem cells; ^*s*^Osteoporosis; ^*t*^Mitogen-activated protein kinase; ^*u*^DNA methyltransferase1; ^*v*^Runt-related transcription factor 2; ^*w*^Alkaline phosphatase; ^*x*^Umbilical cord mesenchymal stem cells; ^*y*^Bone mineral density; ^*z*^Bone volume; ^*aa*^Bone volume/total volume; ^*ab*^Phosphatidylinositol-3-kinases/Protein-serine-threonine kinase.*

## Conclusion and Future Perspectives

Bone is the specific organ for supporting the body, protecting other organs, and storing minerals. Bone disorders normally results in pain and disability, severely reduces the quality of life and increases the burden on society. Therefore, the efficacious therapies for bone diseases have become the major concern worldwide. More recently, MSCs-EV have been shown to mediate the therapeutic effects of MSCs in various diseases and have been applied to clinical trials for several diseases, such as type I diabetes mellitus (trial NCT02138331), macular holes (NCT03437759) and acute ischemic stroke (NCT03384433)^4^. Thus, the role of MSCs-EV in the treatment of bone diseases has become a research focus. Here, we focus on the application of MSCs-EV in four major bone disorders, including OA, RA, OP, and bone fracture.

MSCs-EV, lipid-bilayer spheroids, are characterized by possessing immunomodulatory and regenerative properties, similar to their producing cells. As MSCs-EV mediate both intercellular communication and the interactions with the cellular microenvironments, they have become fascinating to be a novel cell-free therapeutic strategy for treating diseases, including bone disorders. The currently *in vitro* and *in vivo* studies reveal the efficacy of MSCs-EV in treating OA, RA, OP, and bone fracture. MSCs-EV may alleviate the pain, promote overall joint repair and regeneration in OA by suppressing inflammation, promoting cartilage repair and regeneration, and restoring joint homeostasis. They also show immunomodulatory function in RA by regulating the function of T lymphocytes and B lymphocytes and inhibiting angiogenesis via downregulating MMP14 and VEGF, exerting therapeutic effects on joint destruction of RA. Moreover, MSCs-EV improve the osteoporosis by promoting cell proliferation, osteogenic differentiation, and reducing cell senescence of BM-MSCs or osteoblast. Furthermore, the therapeutic effect of MSCs-EV is also observed in bone fracture healing. MSCs-EV orchestrate the process of osteogenesis and angiogenesis to enhance fracture healing. Overall, the present findings suggest MSCs-EV as a new cell-free therapeutic method for bone disorders.

The findings at current stage are very encouraging; however, further research is still needed to make MSCs-EV a routine clinical treatment for bone disease. There are several critical issues to consider, including: (1) The optimal source of MSCs. Since there are many sources of MSCs (e.g., BM-MSCs and AD-MSCs) and MSCs-EV produced by different cell source contain different cargos, it is important to determine the optimal source of MSCs, which may be bioengineered for producing MSCs-EV or their specific cargos according to specific requirements. (2) Standard methods for large-scale production and isolation of MSCs-EV. Because the culture condition affects the MSCs-EV production, the production methods of MSCs-EV should be optimized and controllable for large-scale and specific type of MSCs-EV production. Moreover, there is an urgent need to develop standardized methods for the isolation and purification of MSCs-EV, although establishing a universal isolation method is unlikely due to the complexity of MSC-EV. The isolation methods should not disrupt the structure and functions of MSCs-EV. (3) Methods for rapid and accurate quantification and characterization of MSCs-EV, which are critical for MSCs-EV clinical application. (4) Safe and effective approaches to deliver MSCs-EV to the body or to target sites for treatment. (5) The pharmacokinetics (e.g., tissue distribution, half-life period) of MSCs-EV in the body. (6) Safe and optimal dosage of MSCs-EV for treating different bone diseases. MSCs-exosomes execute synergetic biological functions through combinatorial effects together with their large diverse proteomic and RNA cargo (Lai et al., 2016). It has to be taken into consideration that exosomes do not harbor many copies of miRNA molecules. On average, 100 exosomes would be needed to transfer one copy of a given abundant miRNA. In contrast, MSCs-exosomes proteins in a typical therapeutic dose have the potential to trigger a biologically relevant response (Toh et al., 2018). Moreover, as individual MSCs-EV cargo is not equally or sufficiently efficacious in ameliorating tissue injury (Lai et al., 2012), a safe and optimal dosage needs to be considered. (7) Uncovery of the action mechanisms of MSCs-EV in treating bone disorders by characterizing their functional cargos. Current available publications have demonstrated the involvement of some key molecules and signaling pathways in the therapeutic effects of MSCs-EV on bone disorders, such as Wnts, YAP, AKT, ERK, AMPK, mTOR-autophagy, Wnt/β-catenin, MAPK, and Notch signaling pathways. However, most studies have only identified the mechanism of action of MSCs-EV as a whole, without identifying their specific components. For illustrating the function and mechanism of the MSCs-EV cargo components, current available publications mainly focus on miRNAs. Therefore, the molecular mechanisms of functional components such as MSCs-EV proteins still need to be further investigated. Moreover, current findings show that MSCs-EV exert different effects on angiogenesis in the treatment of RA and bone fracture. MSCs-EV inhibit angiogenesis and the expression of VEGF in treating RA while promote angiogenesis and VEGF expression in treating bone fracture. The molecular mechanism of MSCs-EV is still unclear, further investigation is necessary to make them more suitable for clinical application. (8) The possibility of modifying MSCs and artificially synthesizing MSCs-EV or their specific cargos for treating bone diseases. Biophysical and biochemical methods can be used to modify the properties of MSCs, thereby influencing EV composition and secretion. It leaves a wide range of conditions to be explored in attempts to increase MSCs-EV yields and their therapeutic potential (Park et al., 2019). In addition, understanding the molecular mechanisms of MSCs-EV for the treatment of bone diseases will prevent some side effects and make the clinical application of MSCs-EV more precise and safer. (9) Clinical studies are needed to prove the efficacy of MSCs-EV for the treatment of bone diseased. Clarification of these key issues will make MSCs-EV a more fantastic novel cell-free therapeutic strategy for bone disorders treatment in clinics.

## Author Contributions

SLiu, XX, and SLia drafted the manuscript. ZC and YZ revised the manuscript. SLiu designed the figure. AQ and LH revised and approved the manuscript. All the authors contributed to the article and approved the submitted version.

## Conflict of Interest

The authors declare that the research was conducted in the absence of any commercial or financial relationships that could be construed as a potential conflict of interest.

## Footnotes

1 http://www.exocarta.org

2 http://www.microvesicles.org

3 www.systembio.com

4 www.clinicaltrials.gov

## Abbreviations

β-TCP: β-tricalcium phosphate
AD-MSCs: adipose tissue-derived mesenchymal stem cells
AFM: atomic-force microscopy
AKT: phosphorylation of protein-serine-threonine kinase
ALP: alkaline phosphatase
AMPK: adenosine 5’-monophosphate (AMP)-activated protein kinase
BFR/BS: bone formation rate per bone Surface
BMD: bone mineral density
BM-MSCs: bone marrow mesenchymal stem cells
BV/TV: bone volume/total volume
CIA: collagen-induced arthritis
Col2a1: collagen, type II, alpha 1
Dnmt1: DNA methyltransferase 1
DLS: dynamic-light scattering
ECM: extracellular matrix
ECM/GelMA/exosome: extracellular matrix/gelatin methacrylate/exosome
ERK 1/2: extracellular regulated protein kinases 1/2
ESCRT: endosomal sorting complex required for transport
EV: extracellular vesicles
FLS: fibroblast-like synoviocytes
GAPDH: glyceraldehyde-3-phosphate dehydrogenase
hESC-MSCs: human embryonic-derived mesenchymal stem cells
hiPSC: human-induced pluripotent stem cell
hMSC: human mesenchymal stem cells
HLA-DR: Antigen-antigen D Related
HSP: heat shock proteins
IL-1 α: interleukin-1 α
IL-1 β: interleukin-1 β
ILVs: intraluminal vesicles
IPFP-MSCs: infrapatellar fat pad (IPFP)-derived mesenchymal stem cells
LDHA: lactic dehydrogenase A
lncRNA: long non-coding RNA
MAPK: mitogen-activated protein kinase
MAR: mineral apposition rate
MCP-1: monocyte chemotactic protein 1
MCP-3: monocyte chemotactic protein 3
MHC: major histocompatibility complex
miRNAs: microRNAs
MMP13: matrix metalloproteinase 13
MMP14: matrix metalloproteinase 14
mRNAs: message RNAs
MSCs: mesenchymal stem cells
MSCT: MSCs transplant treatment
mtDNA: mitochondrial DNA
mTOR: mammalian target of rapamycin
MV: microvesicles
MVBs: multivesicular bodies
NTA: nanoparticle-tracking analyses
NGS: next-generation sequencing
OA: osteoarthritis
OP: osteoporosis
PGK1: phosphoglycerate kinase 1
PI3K/AKT: phosphatidylinositol-3-kinases/protein-serine-threonine kinase
PKM: protein kinase
PM: plasma membrane
PCS: photon-correlation spectroscopy
RA: rheumatoid arthritis
ROS: reactive oxygen species
Runx2: Runt-related transcription factor 2
SDF-1: stromal cell-derived factor-1
s-GAG: sulphated glycosaminoglycan
SMSCs: synovial mesenchymal stem cells
Sp1: specificity Protein 1
TGF- β 1: transforming growth factor β 1
TEM: transmission electron microscopy
Th1: T helper cell type 1
Th17: T helper cell type 17
Th2: T helper cell type 2
TMJ-OA: temporomandibular joint osteoarthritis
TNF- α: tumor necrosis factor- α
TRPS: tunable-resistive pulse sensing
uMSCs: umbilical cord mesenchymal stem cells
VEGF: vascular endothelial growth factor
YAP: yes-associated protein.
